# Selection of Effective Therapies Using Three-Dimensional *in vitro* Modeling of Chondrosarcoma

**DOI:** 10.3389/fmolb.2020.566291

**Published:** 2020-12-21

**Authors:** Ieva Palubeckaitė, Sanne Venneker, Inge H. Briaire-de Bruijn, Brendy E. van den Akker, Augustinus D. Krol, Hans Gelderblom, Judith V. M. G. Bovée

**Affiliations:** ^1^Department of Pathology, Leiden University Medical Center, Leiden, Netherlands; ^2^Department of Radiation Oncology, Leiden University Medical Center, Leiden, Netherlands; ^3^Department of Medical Oncology, Leiden University Medical Center, Leiden, Netherlands

**Keywords:** chondrosarcoma, 3D cell culture, chemotherapy, radiotherapy, clonal spheroids, sarcoma, *in vitro* model

## Abstract

**Purpose:** Chondrosarcomas are a group of cartilaginous malignant neoplasms characterized by the deposition of chondrogenic extracellular matrix. Surgical resection is currently the only curative treatment option, due to their high resistance to conventional chemotherapy and radiotherapy. Novel therapeutic treatment options may improve outcome. Predominantly used cell line monolayer *in vitro* models lack *in vivo* complexity, such as the presence of extracellular matrix, and differing oxygen access. Hence, we aimed to improve pre-clinical chondrosarcoma research by developing an alginate-based 3D cell culture model.

**Method:** An alginate scaffold was applied to generate spheroids of three chondrosarcoma cell lines (CH2879, JJ012, SW1353). Morphological, histological and immunohistochemical assessment of the spheroids were used to characterize the chondrosarcoma model. Presto blue assay, morphological and immunohistochemical assessment were applied to assess spheroid response to a panel of chemotherapeutics and targeted therapies, which was compared to conventional 2D monolayer models. Synergistic effect of doxorubicin and ABT-737 (Bcl-2 inhibitor) was compared between monolayer and spheroid models using excess over Bliss. A 3D colony formation assay was developed for assessment of radiotherapy response.

**Results:** Chondrosarcoma spheroids produced chondrogenic matrix and remained proliferative after 2 weeks of culture. When treated with chemotherapeutics, the spheroids were more resistant than their monolayer counterparts, in line with animal models and clinical data. Moreover, for sapanisertib (mTOR inhibitor) treatment, a recovery in chondrosarcoma growth, previously observed in mice models, was also observed using long-term treatment. Morphological assessment was useful in the case of YM-155 (survivin inhibitor) treatment where a fraction of the spheroids underwent cell death, however a large fraction remained proliferative and unaffected. Synergy was less pronounced in 3D compared to 2D. A 3D clonogenic assay confirmed increased resistance to radiotherapy in 3D chondrosarcoma spheroids.

**Conclusion:** We demonstrate that the chondrosarcoma alginate spheroid model is more representative of chondrosarcoma *in vivo* and should be used instead of the monolayer model for therapy testing. Improved selection at *in vitro* stage of therapeutic testing will increase the amount of information available for experimental design of *in vivo* animal testing and later, clinical stages. This can potentially lead to increased likelihood of approval and success at clinical trials.

## Introduction

Chondrosarcomas are a group of malignant cartilaginous neoplasms, which account for around 20% of bone sarcomas, making them the second most common primary bone malignancy after osteosarcoma (Hogendoorn et al., 2013). The hallmark of these tumors is the deposition of extracellular matrix rich in glycosaminoglycans and collagen II. Likeliness of metastasis is best predicted by the histological grade, as an atypical cartilaginous tumor/chondrosarcoma grade I is locally aggressive but rarely metastasizes while grade III chondrosarcomas have a high metastatic rate (32–71%) and poor 10-year survival rate (26–55%) (Angelini et al., 2012; Fromm et al., 2018; van Praag et al., 2018; Bovée et al., 2020). Conventional chemotherapy and radiotherapy have very limited efficacy in patients with advanced chondrosarcoma and surgical resection is currently the only curative treatment option (Angelini et al., 2012; Van Maldegem et al., 2014). Presently there is a large variety in the treatments used in order to attempt to improve survival (van Maldegem et al., 2019). A fraction of patients are managed with drugs which have shown activity in other soft-tissue and bone sarcomas, without any previous pre-clinical information on their efficacy in chondrosarcoma (Italiano et al., 2013). Its rareness, and therefore the lack of interest from industry, hamper the design and conduct of clinical trials for these patients (Miwa et al., 2019). There is an urgent need for more representative pre-clinical chondrosarcoma models to select therapeutic strategies that might be beneficial for patients.

At present, ~10 chondrosarcoma cell lines are available worldwide. These cell lines essentially reflect the heterogeneity of this tumor type, consisting of different subtypes, grades and mutation status (van Oosterwijk et al., 2012a; de Jong et al., 2016a). Cell lines in conventional two-dimensional (2D) monolayer culture can be easily used for high throughput drug screens, and this has revealed several compounds and combinations that might be beneficial for chondrosarcoma patients, including ABT-737 (Bcl-2 inhibitor) in combination with doxorubicin (van Oosterwijk et al., 2012b), YM-155 (Survivin inhibitor) (de Jong et al., 2016b), sapanisertib (mTOR inhibitor) (Addie et al., 2019), as well as radiotherapy (de Jong et al., 2019; Venneker et al., 2019).

However, the limitation of the use of cell lines in standard 2D culture is their restricted representability, as they lack the spatial organization, architecture and extracellular matrix that is characteristic of chondrosarcoma *in vivo* (Bovée et al., 2020). Moreover, pathway activity is altered during culture, for instance we previously showed upregulation of IGF1R expression in CH2879 cell line in 2D culture, while this was not seen in three-dimensional (3D) cultures, nor in the corresponding primary tumor (Peterse et al., 2016). Therefore, cell lines in 2D might not respond to therapy in the way cells do *in vivo*.

A common approach to improve the representability at the *in vitro* testing stage is to create 3D cell culture models. These mimic the natural organization of tissues more closely, which is especially relevant for tumors in which the spatial organization, architecture and interaction with the extracellular matrix are critical. These include chondrosarcoma, where the extracellular matrix is the defining factor, and by some even hypothesized to be involved in chemotherapy resistance (Bovée et al., 2010). Many different methods exist for the creation of 3D cell cultures of sarcoma including attachment prevention, embedded scaffold and bioreactor techniques. Each has advantages and disadvantages for sarcoma research (Colella et al., 2018).

To develop a 3D spheroid culture model for chondrosarcoma, we chose to use a biocompatible, plant derived scaffold, alginate hydrogel (Lee and Mooney, 2012; Lhuissier et al., 2017). Alginate hydrogel polymerises rapidly in the presence of divalent ions, such as Ca^2+^, therefore can be used for entrapment of single cells, which subsequently grow out into clonal spheroids. As the cells are embedded within a scaffold system, they receive physiological mechanical strain, are able to grow in three dimensions and deposit extracellular matrix (ECM) which will then remain within their surroundings. Alginate scaffolds are also suitable for drug treatment of cultures, as the pore size achieved is typically 5–200 nm and small soluble molecules are able to diffuse freely (Ching et al., 2017).

In this study, alginate chondrosarcoma spheroids were shown to produce extracellular matrix, similar to tumors *in vivo*, and were used for short and long-term drug testing, including assessment of synergy. Drug response within the model was monitored using cell viability assays as well as histological analysis and the model was additionally used to evaluate radiation sensitivity using a 3D colony formation assay.

## Materials and Methods

### Cell Culture

CH2879 (Grade III, *IDH1*: G105G (wt), *p53*: wt) (Gil-Benso et al., 2003), JJ012 (Grade II, *IDH1*: R132G mut, *p53*: G199V mut) (Jagasia et al., 1996; Scully et al., 2000) and SW1353 (Grade II, *IDH2*: R172S mut, *p53*: V203L mut) (ATCC, Manassas, USA) conventional chondrosarcoma cell lines were cultured in RPMI 1,640 medium (Life Technologies Limited, Paisley, UK) containing 10% fetal bovine serum (FBS) and 50 U/ml penicillin and 50 μg/mL streptomycin (Life Technologies Limited) (Pansuriya et al., 2011; de Jong et al., 2016b). They were maintained in a humidified atmosphere containing 5% CO_2_ at 37°C. Short tandem repeat analysis was performed before and after completion of experiments to confirm identity of the cell lines by using the Cell ID Gene Print 10 system (Promega Benelux BV, Leiden, The Netherlands). Mycoplasma negativity was confirmed on a regular basis. The SJSA-1, also referred to as OSA, (ATCC) osteosarcoma cell line was also cultured identically for use as a negative control for histological stains.

### 3D Cell Culture

Following expansion in a monolayer, cell lines were suspended in 1.2% (w/v) medium viscosity alginic acid (Sigma-Aldrich, Darmstadt, Germany) in 0.15 M NaCl at 1 × 10^6^ cell/mL density to generate spheroids (~14,140 cells/bead). Concentrations of around 1% w/v alginate were deemed optimal for cartilage-orientated *in vitro* and *in vivo* applications by several groups (Heiligenstein et al., 2011; Rey-Rico et al., 2016). Alginate beads were formed by dropping the cellular suspension in 1.2% (w/v) alginate/0.15 M NaCl (Sigma-Aldrich) through a 19-gauge needle into 0.2 M CaCl_2_ solution (Sigma-Aldrich). After incubation at 37°C for 10 min, alginate beads were washed twice with 0.15 M NaCl and washed twice in DMEM/F12 (Life Technologies Limited) complete media before being placed in RPMI medium overnight. The medium was replaced to DMEM/F12 medium where possible due to the alginate breakdown properties of phosphate buffer within RPMI medium (Lee and Palsson, 1990). The transition from RPMI to DMEM culture was made on the second day of culture in 3D to minimize transitioning stress on the cells. Preliminary tests were performed to determine the best time to transition the medium; before, during or after 3D cell culture establishment. Changing medium to DMEM 24 h after 3D cell culture was found to be the least disturbing for cell growth. The cells were cultured in DMEM/F12 for a maximum of 17 days.

### Histological Staining

Day 3, 7, and 14 spheroids were fixed using 10% neutral buffered formalin, dehydrated and paraffin embedded. Sections were produced (4 μm) and, after deparaffinisation and rehydration, were stained with hematoxylin and eosin (H&E), using the Leica ST5020-CV5030 Stainer Integrated Workstation (Leica Biosystems, Amsterdam, The Netherlands), for morphological assessment by an expert bone tumor pathologist (JVMGB). Serial sections were stained with Richardson's toluidine blue or alcian blue solution, for assessment of glycosaminoglycan production; slides were incubated in boiling 10 mM citrate buffer (pH 6) for 10 minutes in order to remove the alginate scaffold, which is stained very strongly with both stains. After cooling and washing in dH_2_O, sections were incubated in toluidine blue solution (1:32 diluted in dH_2_0) for 5 min or incubated (30 min) in alcian blue solution (pH 2.5, RT). Excess dye on toluidine blue stained samples was removed in dH_2_O and the slides were dried at 58°C for 30 min or until fully dry, before mounting. Alcian blue stained samples were washed in running tap water (2 min), rinsed in dH_2_O, counterstained with nuclear fast red (5 min), dehydrated, cleared and mounted. SJSA-1 cell line spheroids were stained as a negative control. For comparison to 2D, cell lines cultured in 2D were trypsinised, formalin fixed (30 min) and pre-embedded using the cytoblock system (7401150, Thermo Scientific, USA). Then the samples were dehydrated and paraffin embedded for sectioning. For comparison to chondrosarcoma *in vivo*, three high grade chondrosarcomas were retrieved from the archives of the Department of Pathology, Leiden University Medical Center, Leiden, the Netherlands. Tumor samples were fully anonymized according to the ethical guidelines described in “Code for Proper Secondary Use of Human Tissue in The Netherlands” of the Dutch Federation of Medical Scientific Societies.

### Immunohistochemistry

Four micrometer paraffin embedded sample sections were deparaffinized in xylene and rehydrated with a series of gradient ethanol to water. For Ki-67 [anti-Ki-67 D2H10 rabbit monoclonal (1:1600)] (9027S, Cell Signaling, Massachusetts, USA), IGF1R [anti-IGF1R 111A9 rabbit monoclonal (1:2000)] (3018, Cell Signaling), activated caspase-3 [anti-activated caspase-3 rabbit polyclonal (1:1600)] (9661S, Cell Signaling), and phospho-S6 [anti-phospho-S6 ribosomal (2F9) rabbit monoclonal (1:400)] (4856S, Cell Signaling) detectionheated antigen retrieval was performed by incubating slides in boiling 10 mM citrate buffer (pH 6) for 10 min and letting the solution cool down for 2 h. For Collagen type II [anti-collagen II rabbit polyclonal (1:200)] (34712, Abcam, Cambridge, United Kingdom) detection enzymatic antigen retrieval was performed via incubation in proteinase K (5 μL/mL) (3 min, RT) (Roche) followed by an incubation with hyaluronidase (5 mg/mL) (Sigma-Aldrich) (30 min, 37°C). Endogenous peroxidase was blocked by pre-incubation in H_2_O_2_ (0.3%) for 20 min and 5% non-fat milk solution was additionally used for the anti-collagen II primary antibody. Slides were incubated with the primary antibodies overnight at 4°C. Slides were incubated in biotin-free poly-HRP anti-Mouse/Rabbit IgG secondary antibody for 30 min (VWRKDPVO110HRP, VWR International, Amsterdam, The Netherlands). The signal was then visualized by incubation in DAB+ Chromogen for 10 min (K3468, Dako, Carpinteria, USA). The slides were counterstained in hematoxylin for 20 s, dehydrated and mounted for scanning using a Pannoramic 250 Flash III scanner (3D Histech, Cheshire, UK) using a 40× magnification. Positive and negative controls were used for each antibody for quality control of the experiment.

The scanned IHC samples were scored using QuPath open-source software (Bankhead et al., 2017). All scoring was performed on a representative area spanning at least three biological replicates (beads). Scoring of Ki-67 was performed using the software positive cell detection tool to determine the percentage of positive nuclei among the total number of nuclei within an area larger than 800,000 μm^2^. Scoring of activated caspase-3 staining was performed by manual counting of percentage of positive cells within an area larger than 600,000 μm^2^. Scoring of IGF1R was performed as previously published for 2D cells and tissue, based on overall intensity within an area larger than 700,000 μm^2^ (Peterse et al., 2016). Scoring of collagen II and pS6 was performed by scoring slides by percentage of positive spheroids within an area larger than 900,000 μm^2^. In this case a spheroid was termed positive if at least one area within the spheroid was calculated positive using the software positive cell detection tool. Setting the software detection threshold ensured reduction in observation variability. Samples were collected over at least three separate experiments and areas counted were from at least three replicates.

### Assessment of Response to Antineoplastic Agents

Dose-response curves were generated for the three cell lines in 2D as well as 3D, keeping within 15 passages throughout the experiment. After trypsinisation, 2D cultured cells were seeded in 96-well plates at 7,000 (CH2879) or 3,000 (JJ012, SW1353) cells per well and left to adhere for 24 h. Treatment duration for 2D cultures was 72 h. In the case of 3D cell cultures, day 14 spheroids were used for 72 h treatment measurements and day 1 spheroids were used for 14 day treatments, including a measurement at 8 days (after 7 days of treatment) and a second dose at day 8.

Compounds were selected based on previous results (van Oosterwijk et al., 2011; de Jong et al., 2016b, 2018, 2019; Addie et al., 2019; Venneker et al., 2019). Doxorubicin and cisplatin were obtained from the in-house hospital pharmacy in a 0.9% (w/v) NaCl solution. ABT-737 (Bcl-2 inhibitor) (S1002, Selleckchem, Houston, TX, USA), AGI-5198 (mutant-IDH1 inhibitor) (14624, Cayman Chemical, Ann Arbor, MI, USA) YM155 (survivin inhibitor) (S1130, Selleckchem) and sapanisertib (mTOR inhibitor) (S2811, Selleckchem) were dissolved in DMSO according to the manufacturer's instructions.

After treatment, the medium was removed, wells were washed with medium and cell viability was determined using the PrestoBlue viability reagent (Invitrogen, Life-Technologies, Scotland, UK) according to the manufacturer's instructions. Plates were then incubated at 37°C for 1 h for monolayer cultures, one and a half hours for CH2879 and JJ012 spheroid cultures and 3 h for SW1353 spheroid cultures. Fluorescence was measured with an excitation/emission wavelength of 560/590 nm Victor3V 1420 multilabel counter (Perkin Elmer, Hamburg, Germany). Selected treated samples were processed for sectioning and histological and IHC staining.

Histological staining was also performed on select treated cultures. These were stained with H&E and the colony number was counted within a representative area spanning at least three biological replicates (beads).

The drug concentration which inhibited cell viability by 50% (IC_50_) was determined using Prism software version 7.02 after normalization to untreated control samples. A Grubbs' test was used to remove outliers from the dataset. Experiments were performed at least three times in triplicate. A Welch's ANOVA test was used to determine significant differences between the IC_50_ values obtained from 2D and 3D cultures. A *post-hoc* Dunnett's T3 multiple comparisons test was then performed for comparison of individual cell line results in 2D and 3D.

### Assessment of Drug Synergy

Increasing concentrations of doxorubicin were used to treat the chondrosarcoma spheroid cultures in combination with 3.16 μM ABT-737 as this drug combination has demonstrated synergy in previously published monolayer models (van Oosterwijk et al., 2012b). The Bliss independence model (C = A + B – A × B) was used to predict synergy, in which C represents the combined effect and A and B represent single agent effects (Borisy et al., 2003).

### Assessment of Radiation Therapy Response

One day old spheroids were used for radiation treatment experiments. Five alginate beads per well were transferred to a 12-well plate. Increasing doses (0–6 Gy) of γ-radiation, using a ^137^C source (YXLON, Comet Technologies, Shelton, USA), were applied to culture plates containing cell seeded alginate beads. These were cultured for 14 days, with a medium refresh at 7 days, formalin fixed using 10% formalin, dehydrated and paraffin embedded. The samples were then sectioned, stained using H&E, and the spheroid colony number and area, within an area of 3 mm^2^, was assessed using QuPath open source software (Bankhead et al., 2017). All counts were performed on a representative area spanning at least three biological replicates (beads).

## Results

### Chondrosarcoma Cells Cultured Within an Alginate Model Display a Phenotype Closer to That of Chondrosarcoma *in vivo*

**Figure 1:**
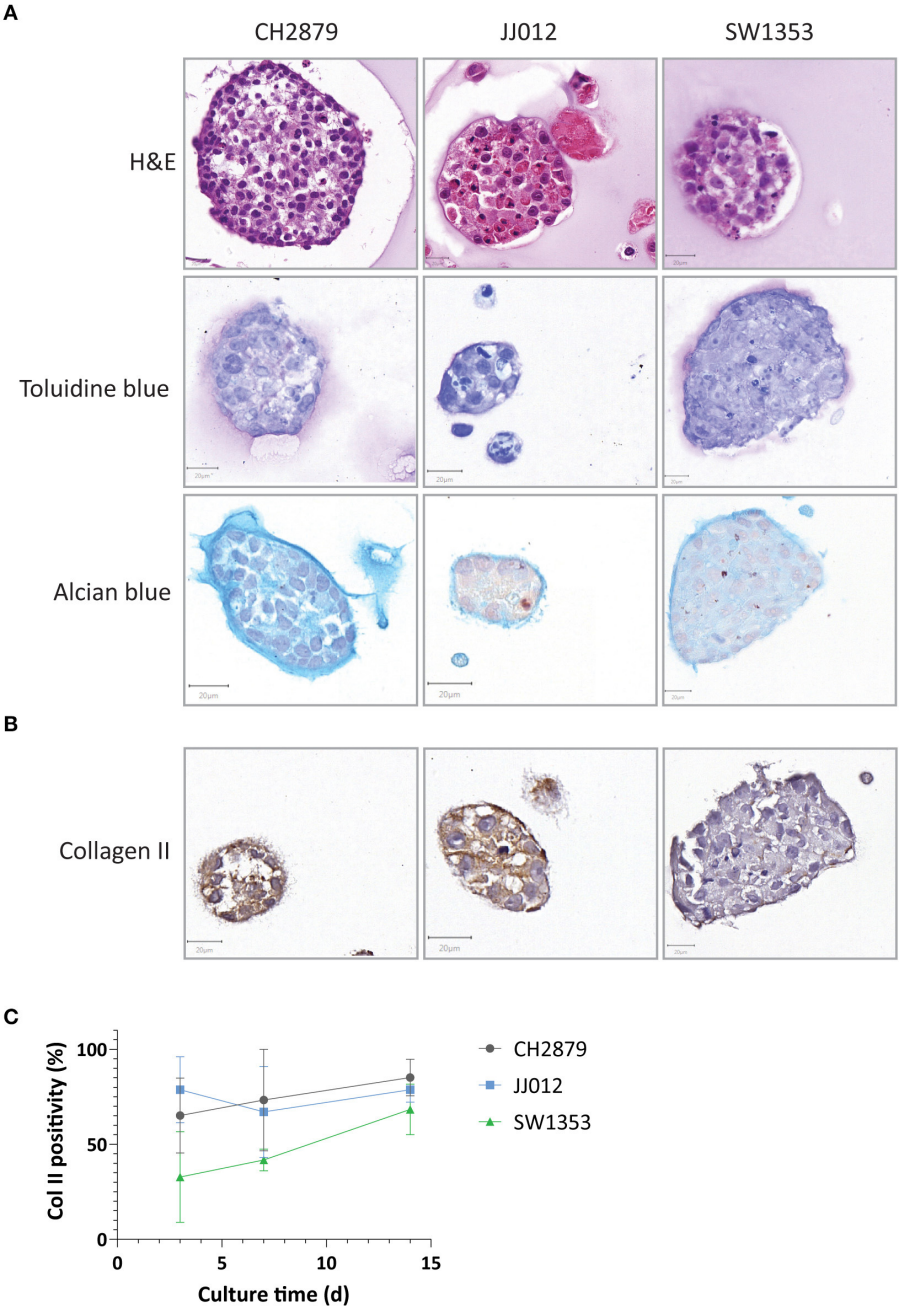
Chondrosarcoma 14 day alginate spheroid cultures were assessed for **(A)** Spheroids presented chondrosarcoma-like histology (as seen by HandE) and were positive for chondrosarcoma extracellular matrix components such as glycosaminoglycans (toluidine and alcian blue staining) **(B)** Collagen II expression was present in all three cell lines and **(C)** expression levels remained stable or increased over time (*n* ≥ 3). Scale bar = 20 μm.

All cell lines cultured in alginate beads formed chondrosarcoma spheroids ranging from 50 to 156 μm diameter by 14 days of culture with an average diameter of 76 μm. All spheroids displayed extracellular matrix deposition, as confirmed by toluidine blue and alcian blue staining (Figure 1A), while the osteosarcoma cell line (SJSA-1) spheroid that served as control was negative as well as the cell lines cultured in 2D (Supplementary Figures 1, 2). Glycosaminoglycans were deposited throughout the spheroids and concentrated around the edges. Thus, in the alginate model, chondrosarcoma cells show some differentiation, as they produce extracellular matrix, similar to chondrosarcoma *in vivo* (Supplementary Figure 1). Collagen II was also expressed in the extracellular matrix and was stable or increased over time (Figures 1B,C). These features are hallmarks of chondrosarcoma but known to be absent in 2D culture (Scully et al., 2000; Gil-Benso et al., 2003; Gebauer et al., 2005) which we confirmed as the three cell lines cultured in 2D lacked glycosaminoglycans and collagen II expression, while expression was abundant in the three high grade chondrosarcoma tissue specimens (Supplementary Figure 1).

**Figure 2:**
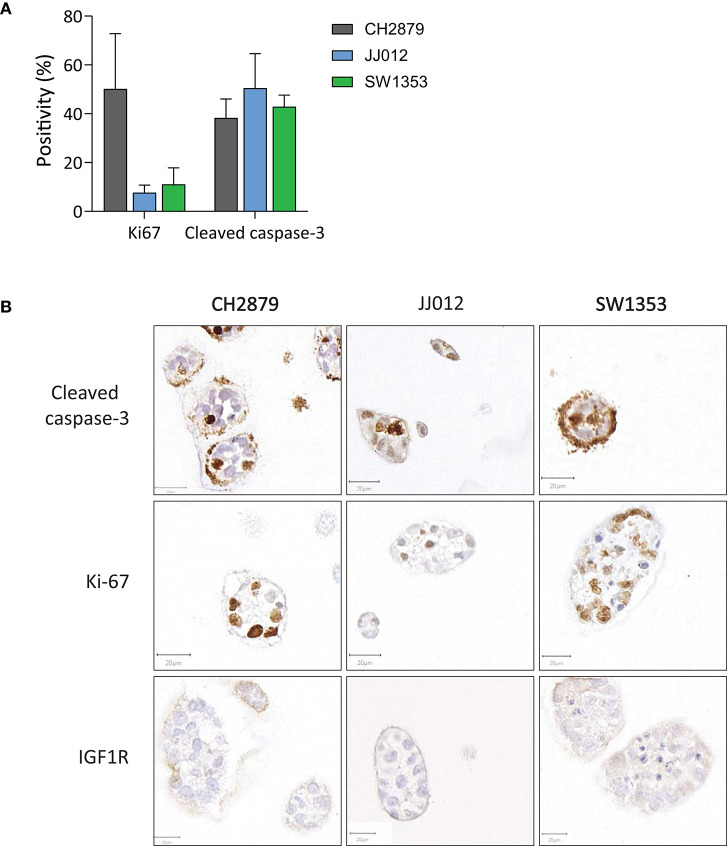
**(A)** Quantification of apoptosis (cleaved caspase 3) and proliferation (Ki-67) in the spheroids showed that on average <50% of cells were apoptotic. Proliferation was observed in all cell lines, and was particularly high for CH2879. **(B)** Apoptosis (cleaved caspase 3), proliferation (Ki-67) and IGF1R expression patterns varied within spheroid cultures. IGF1R was not expressed (*n* ≥ 3). Scale bar = 20 μm.

CH2879 exhibited high proliferative activity as shown by Ki-67 immunohistochemistry at 14 days of 3D culture (50%), whereas a smaller portion of JJ012 and SW1353 cultures was proliferative at this stage (8 and 11%) (Figures 2A,B). In comparison, the average proliferative activity (22.9%) was more similar to that of chondrosarcoma tissue (25.9%) than 2D cultured cell lines (82.7%) (Supplementary Figure 3). Cleaved caspase-3 levels in all three cultures were relatively high (total average = 43.9%), indicating a high level of apoptosis within the 3D cultures. For comparison, the apoptotic levels in chondrosarcoma tissue were higher than in 2D cell cultures (3.2% compared to 0.3% average) but not at similar levels to our model (Supplementary Figure 3). Since we previously reported IGF1R expression to be artificially upregulated in chondrosarcoma cell lines, being low to absent in chondrosarcoma primary tumor tissue but positive in 2D cultured cell lines (Peterse et al., 2016), we assessed IGF1R in the spheroids. IGF1R expression was negative in all samples (Figure 2B), with an adequate external positive control (Supplementary Figure 4).

### Chondrosarcoma Spheroids Respond Differently to Antineoplastic Agents as Compared to Monolayers

**Figure 3:**
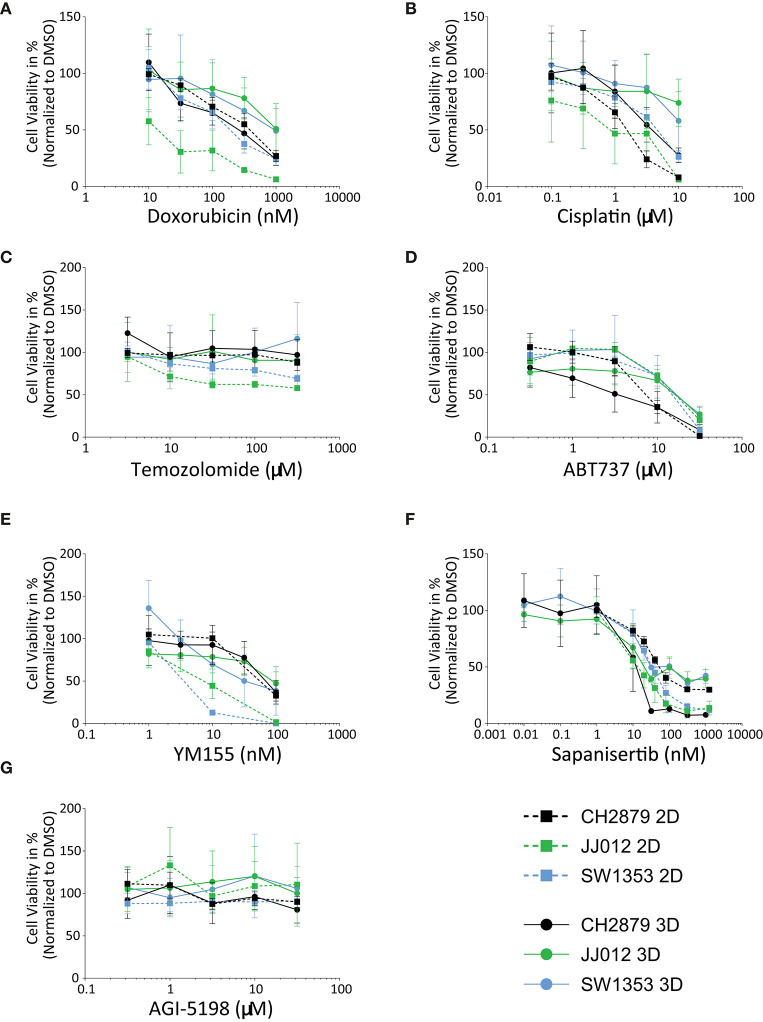
Chondrosarcoma cell lines CH2879, JJ012, SW1353, cultured in 2D or 3D spheroid conditions, responded variably when treated with a panel of antineoplastic drugs; **(A)** Doxorubicin, **(B)** Cisplatin, **(C)** Temozolomide, **(D)** ABT-737, **(E)** YM-155, **(F)** Sapanisertib, **(G)** AGI-5198. For most compounds, cells in 2D are more sensitive as compared to 3D, with the exception of CH2879 when treated with doxorubicin or sapanisertib, CH2879 and JJ012 when treated with ABT-737- (*n* ≥ 3). All results were normalized to DMSO/PBS control as applicable.

In general, the 3D *in vitro* cultures were more resistant to the panel of tested compounds, as can be seen from the resulting IC_50_ values and 3D/2D ratios (Table 1). All three cell lines were more resistant to doxorubicin (JJ012 3D/2D = 56.5, SW1353 3D/2D = 3.7) and cisplatin (CH2879 3D/2D = 2.9, JJ012 3D/2D = 21.6, SW1353 3D/2D = 3.8) (with the exception of CH2879 treated with doxorubicin, 3D/2D= 0.7) (Figures 3A,B). During temozolomide treatment, all three cell lines showed more resistance in spheroid as compared to monolayer culture (Figure 3C). CH2879 and JJ012 spheroids were more sensitive to ABT-737 treatment (CH2879 3D/2D = 0.4, JJ012 3D/2D = 0.7) whereas SW1353 spheroids were more resistant (3D/2D= 1.4) (Figure 3D). YM-155 treatment followed a similar trend to most treatments, with higher resistance to drug for all spheroid lines (CH2879 3D/2D = 1.2, JJ012 3D/2D = 10.1, SW1353 3D/2D = 11) (Figure 3E).

**Table 1:** IC_50_ values for 2D and 3D cultured chondrosarcoma cell lines treated with a small panel of compounds.

	**2D IC**_****50****_			**3D IC**_****50****_			**3D/2D ratio**		
**Compound**	**CH2879**	**JJ012**	**SW1353**	**CH2879**	**JJ012**	**SW1353**	**CH2879**	**JJ012**	**SW1353**
Cisplatin (μM)	1.48 ± 0.16	1.05 ± 0.26	4.08 ± 0.28	4.26 ± 0.68 (^****^)	22.64 ± 7.26 (^****^)	15.30 ± 4.28 (^****^)	2.9	21.6	3.8
Doxorubicin (nM)	334.6 ± 22.63	17.72 ± 2.87	200.2 ± 22.12	230.5 ± 32.92 (^****^)	1002 ± 229.3 (^****^)	750.6 ± 182.8 (^****^)	0.7	56.5	3.7
Temozolomide (μM)	2390 ± 267.9	188.8 ± 32.82	523.8 ± 63.21	n/e	2470 ± 2176 (^**^)	n/e	resistant 3D	13.1	resistant 3D
ABT-737 (μM)	7.95 ± 1.31	18.93 ± 3.45	14.35 ± 1.99	3.26 ± 0.57 (^****^)	13.42 ± 2.22 (^***^)	19.82 ± 5.08 (^*^)	0.4	0.7	1.4
YM-155 (nM)	66.12 ± 9.74	7.22 ± 1.15	3.66 ± 0.65	76.72 ± 13.69 (ns)	73.27 ± 12.25 (^****^)	40.37 ± 10.64 (^****^)	1.2	10.1	11.0
Sapanisertib (nM)	62.23 ± 5.73	15.64 ± 1.34	35.08 ± 2.02	13.52 ± 2.82 (^****^)	75.74 ± 16.14 (^****^)	113.9 ± 24.92 (^****^)	0.2	4.8	3.2
AGI-5198 (μM)	n/e	n/e	n/e	n/e	n/e	n/e	resistant	resistant	resistant
Dox + ABT (nM)	128.7 ± 8.11	13.36 ± 2.11	80.12 ± 6.94	68.86 ± 9.33 (^****^)	1092 ± 293.2 (^****^)	1167 ± 384.7 (^****^)	0.5	81.7	14.6

The 3D/2D ratio is also given representing the difference in IC_50_ between 2D and 3D cultures. Ratio values of less than one indicate factor of higher sensitivity in 3D culture (red), values above one indicate a higher drug resistance in 3D spheroids (green) and no difference in result = 0 (yellow). If a line is unaffected by the treatment in both 2D and 3D culture it is labeled resistant, if only unaffected in 3D culture it is labeled resistant 3D. Significance was determined between 2D culture IC_50_ and the 3D culture counterparts. Significance is interpreted as 0.1234 (ns), 0.0332

(*) , 0.0021

(**) , 0.0002

(***) , < 0.0001

(****) *on the 3D IC_50_ columns*.

*n/e= no effect*.

CH2879 spheroids were more sensitive to sapanisertib compared to monolayer cultures (3D/2D ratio= 0.2), while for JJ012 and SW1353 spheroids were more resistant (JJ012 3D/2D = 4.8, SW1353 3D/2D= 3.2) (Figure 3F). AGI-5198, a mutant-IDH1 inhibitor, had no significant effect on the cell lines in monolayer nor spheroids (Figure 3G).

### Treatment Response at the Histological Level Is Highly Variable

**Figure 4:**
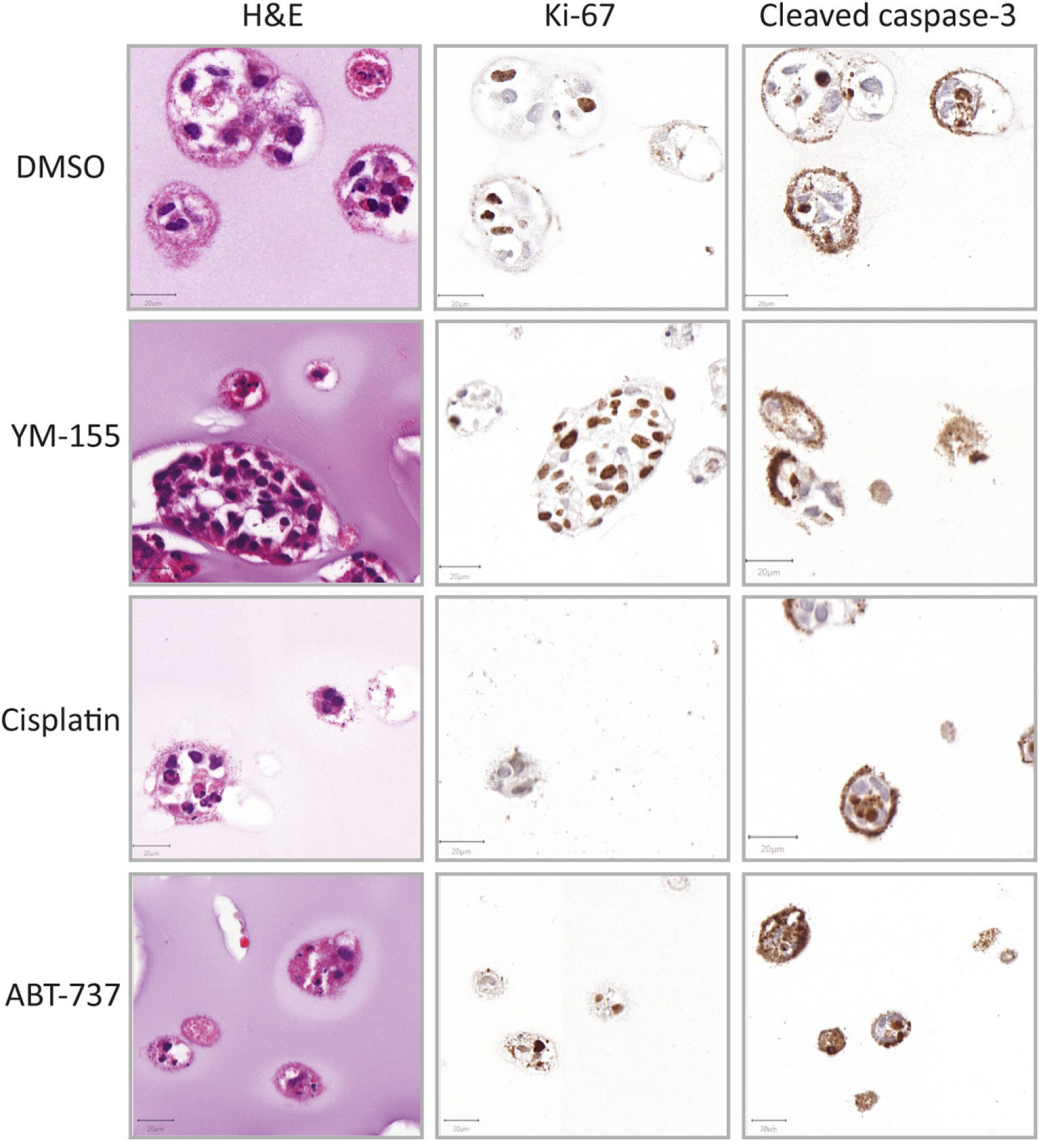
Histological evaluation after treatment (CH2879). Morphology (HandE), level of proliferation (Ki-67) and apoptosis (cleaved caspase-3) were evaluated after treatment (YM-155 = 100 nM, cisplatin = 10 μM, ABT-737 = 31.6 μM). YM-155 treatment indicated presence of smaller, treatment affected, as well as larger, unaffected, proliferative spheroids. Cisplatin treatment resulted in presence of smaller spheroids as well as global cell death and ABT-737 treatment induced apoptosis (*n* = 1). Scale bar = 20 μm.

Additionally to the global cell viability dose response curve data, histological analysis of treated spheroids revealed further information on different morphological effects on the spheroids. CH2879 spheroids are displayed as an example of drug effect (Figure 4). YM-155 treatment showed a reduction in number and clear loss of viability in smaller diameter spheroids, whereas larger spheroids remained proliferative (Figure 4, Supplementary Figure 5). On the other hand, cisplatin treated spheroids showed a more global chemotherapy effect, with an overall decrease in spheroid size and number. The majority of ABT-737 spheroids were showing signs of apoptosis (shrunken nuclei and cleaved caspase-3 positivity) (Figure 4).

### CS Spheroid Model Can Be Used to Evaluate Synergy

**Figure 5:**
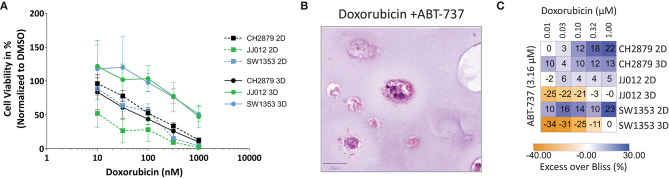
Evaluation of synergy. **(A)** Combination treatment with doxorubicin and ABT-737 was shown to be more effective in 2D than 3D cultures (*n* = 3). **(B)** The number and size of colonies was reduced after treatment. Scale bar = 20 μm, cell line = CH2879. **(C)** Synergy heatmap indicating Excess over Bliss values for chondrosarcoma *in vitro* combination therapy shows that spheroids lack the synergy that is seen using monolayer cells. Synergistic > 0, additive = 0, antagonistic < 0. Results were normalized to DMSO control.

Chondrosarcoma spheroids were also utilized for prediction of synergy. As demonstrated by combining doxorubicin and ABT-737 the excess over Bliss values, indicating synergy, also differ between the monolayer and spheroid cultures (Figure 5). The combination of Bcl-2 inhibition using ABT-737 and doxorubicin was previously shown to reduce cell viability *in vitro* by inducing apoptosis and overcoming chemoresistance (van Oosterwijk et al., 2011). Synergy is less pronounced in CH2879 spheroids compared to 2D (Excess over Bliss= 22 vs. 13%) and combination was only additive for JJ012 (Excess over Bliss = 5 vs. 0%) and SW1353 (Excess over Bliss = 23 vs. 0%) spheroids (Figure 5C). Although around 10% of total spheroids were still viable, as indicated by presto blue assay, even at 1 μM doxorubicin; histological analysis showed a higher amount of cell death in remaining spheroids for all three cell lines compared to single-agent options (Figures 3A,D, 5A,B).

### CS Spheroid Model Can Be Used for Long-Term Treatment

**Figure 6:**
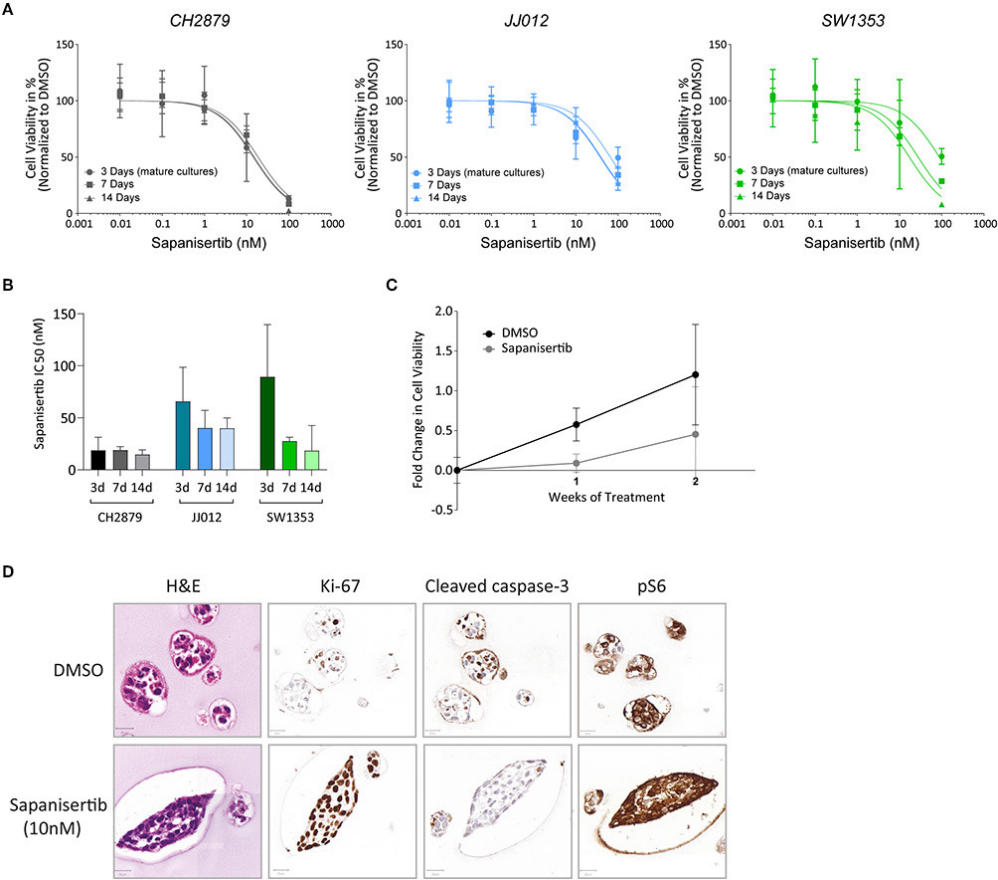
Long-term treatment. **(A)** There is no significant difference in the viability of cell lines CH2879, JJ012 and SW1353 cultured in 3D if they receive 3 days of sapanisertib treatment as mature spheroids or treated over their growth period for 7 days or 14 days (doses at days one and eight). (*n* ≥ 3) **(B)** There is no significant difference in the IC50 values between mature spheroid 3 day treatment or treatment over the growth period. **(C)** Growth rate of CH2879 spheroids treated with sapanisertib (10 nM) increased over 2 weeks. (*n* = 4) **(D)** No significant change in colony morphology or number was observed after 10 nM treatment. Cells after 2 weeks of sapanisertib treatment were highly proliferative while apoptosis was absent and expression of pS6 was uninhibited, suggesting outgrowth of resistant colonies over time. Scale bar = 20 μm, cell line = CH2879. All results were normalized to DMSO control as applicable.

Sapanisertib, an mTOR inhibitor, was initially used for 3 day treatment of mature 14 day old spheroids. All three cell lines were sensitive to the treatment, similar to previously published 2D results (Table 1) (Figure 3F) (Addie et al., 2019). For long-term treatment, two doses of sapanisertib were given overall throughout spheroid growth, one at day one and one at day eight. Cell viability response measured at 7 day and 14 day treatment with sapanisertib did not significantly differ from response to 3 day treatment of mature spheroids for any of the three cell lines tested (Figure 6A), however a trend toward higher resistance to sapanisertib in two out of three mature cultures was observed (Figure 6B). These results show that our model is suitable for up to 14 days of treatment, but that in case of sapanisertib, increase in treatment length and number of doses do not lead to lower cell viability. CH2879 fold change in cell viability at 10 nM sapanisertib treatment shows an initial delay in growth at 1 week of treatment compared to control culture followed by an increase in growth rate by 2 weeks, which may suggest outgrowth of a resistant cell population (Figure 6C). Similar trends were observed with the other two cell lines (Supplementary Figure 6). This was confirmed by histological analysis which indicated presence of unaffected, slightly more proliferative spheroids in the treated group (65.63% Ki-67 positive cells compared to 52.32%) (Figure 6D). The remaining spheroids were also shown to have normal expression of pS6, the activated form of a kinase downstream of mTOR, after mTOR inhibition (Figure 6D).

### CS Spheroid Model Allows for Direct Assessment of Radiation Response

**Figure 7:**
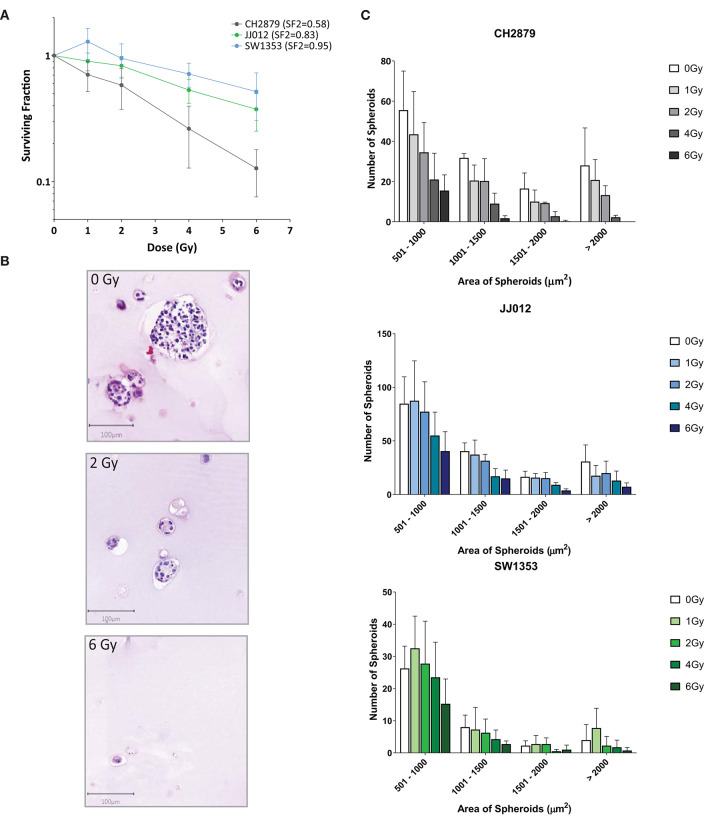
Chondrosarcoma spheroids were irradiated with indicated doses and response evaluated by 3D colony formation assay (*n* = 4). **(A)** All three cell lines were sensitive to radiation at higher dosages (>2Gy), however CH2879 also showed sensitivity at lower doses (SF2 = 0.58). **(B)** CH2879 spheroid colony number and size decrease with increased radiation dose **(C)** Spheroid area (size) was also reduced with increased dose.

Colony formation assays are typically employed to observe sensitivity to radiotherapy (Franken et al., 2006). This method is dependent on the cell clonogenic capacity, which is reduced after irradiation. As the spheroids produced using our method are clonal and cells cannot migrate freely within the scaffold, we adapted the colony formation assay for 3D. Significant changes in the surviving fraction after 2Gy (SF2) treatment are considered indicative of the *in vivo* response to radiotherapy (Fertil and Malaise, 1981). CH2879 spheroids appear to be the most sensitive to radiotherapy (SF2 = 0.58) and JJ012 and SW1353 spheroids only have a small reduction in clonogenic capacity (SF2 = 0.83 and 0.95, respectively) (Figure 7B).The number of spheroids as well as their size is reduced with increased radiation dose for all three chondrosarcoma cell lines, with CH2879 shown as a histological example (Figures 7A,B). Size of colonies as well as surviving fraction is reduced with increased irradiation (Figure 7C).

## Discussion

Although cell lines cultured in monolayer are easy to work with, they do not adequately mimic the morphology and the behavior of tumor cells in patients (Horvath et al., 2016). For rare cancers, representative *in vitro* models are especially needed in order to adequately predict which compounds could provide patient benefit, as organizing clinical trials for rare cancer patients is challenging. In this paper we developed a more representative model for conventional chondrosarcoma of bone.

To date, chondrosarcoma cell lines have been cultured in 3D using non-adherent surfaces (van Oosterwijk et al., 2012b), the hanging drop method (Perut et al., 2018), collagen scaffolds (Truong et al., 2012; Hamdi et al., 2015) and alginate beads (Lhuissier et al., 2017). The attachment prevention techniques used succeeded in formation of large regular multicellular tumor spheroids (MCTS) with corresponding representative regions of quiescence, hypoxia and necrosis, whereas cells within the collagen gel model migrated throughout the gel and oxygen levels were regulated externally in order to mimic *in vivo* hypoxic circumstances (van Oosterwijk et al., 2012b; Hamdi et al., 2015). Use of scaffolds such as alginate, containing a mechanical strain component but where migration is limited, may be beneficial for the 3D culture of sarcoma.

Morphological features similar to high grade chondrosarcoma could be observed from H&E stained spheroids and extracellular matrix components were highlighted using histological and immunohistochemical means, allowing for a suitable comparison to observations in patients. The straightforward application of methods conventionally used to assess patient tissue samples is a major advantage of 3D culture over 2D models. The development of 3D cultures for mesenchymal tumors requires a different approach as compared to the more frequent epithelial cancers, as many sarcomas are characterized by the deposition of extracellular matrix, including collagen, hyaluronan and glycoproteins. Thus, 3D sarcoma models should facilitate matrix deposition by the tumor cells, over a short culture period. Presence of glycosaminoglycan and collagen II in all chondrosarcoma cell line spheroids confirmed the deposition of a chondroid matrix, which increases model similarity to *in vivo* (Aigner et al., 1997). Moreover, levels of IGF1R, that we previously showed to be artificially upregulated during culture in 2D, were negative in the spheroids, also indicating a higher similarity to chondrosarcoma *in vivo* (Peterse et al., 2016). Culture induced upregulation of genes or pathways will affect drug response, and use of the spheroid model will reduce the number of artificially upregulated targets.

Of note, there was a high level of apoptosis observed within the spheroids, which may be a reflection of tissue remodeling related programmed cell death, which is normal for a large cell mass. This was also reported by others (Lhuissier et al., 2017). The alginate scaffold is highly biocompatible and is therefore unlikely to be the cause of apoptosis, as it has been previously used for many cell-based applications (Andersen et al., 2015). Moreover, cells are unable to migrate through the alginate, and dead cells or debris would remain trapped. All of the cultures maintained proliferation at 14 days of culture at similar levels to *in vivo* on average, indicating a sufficient continuation of cell growth during the therapeutic treatment period, and we showed that long-term treatment is feasible.

Overall, response to chemo- and radiation therapy in 3D chondrosarcoma spheroids were more in line with results *in vivo* as compared to monolayer (Table 2). Generally, higher resistance to classical chemotherapeutics, such as doxorubicin, cisplatin and temozolomide was observed. These results are in line with current *in vivo* and clinical data (Table 2) as conventional chemotherapy is not effective as single-agent treatment for chondrosarcoma (Skubitz, 2003; Italiano et al., 2013; van Maldegem et al., 2019). The increased resistance in 3D might be explained by the presence of matrix components, pH and oxygen gradients as well as lowered exposure to therapy within spheroid cultures compared to monolayer, where all cells are equally fully exposed. There were also some exceptions where response in 3D and monolayer did not significantly differ, such as doxorubicin treated CH2879.

**Table 2:** Summary of the sensitivity of 2D, 3D *in vitro*, mouse *in vivo* conventional chondrosarcoma models to selected chemo-/radio-/targeted therapies.

**Compound**	**Cell lines 2D**	**Cell lines 3D**	**Animal model**	**Patient data**	**Ongoing clinical trials**
Cisplatin	Sensitive^*^ (de Jong et al., 2018)	More resistant^*^ (Lhuissier et al., 2017)	Not performed	Considered resistant. Used in combination with doxorubicin, never as single agent. Increased PFS associated with combination treatment (*n* = ?/21^†^, *n* = 4) (Italiano et al., 2013; van Maldegem et al., 2019)	Combination of cisplatin with FT 2102 recruiting (NCT03684811)
Doxorubicin	Sensitive^*^ (de Jong et al., 2018)	JJ012, SW1353 more resistant, CH2879 slightly more sensitive^*^	Resistant (Perez et al., 2012; van Oosterwijk et al., 2015)	Efficacy low in conventional chondrosarcoma, even when using liposomal version. PFS longer when combined with other agents (*n* = ?/32^†^, *n* = 2, *n* = 1) (Skubitz, 2003; Italiano et al., 2013; van Maldegem et al., 2019)	Combination of doxorubicin with nilotinib ongoing (NCT02587169)
Temozolomide	JJ012 and SW1353 sensitive, CH2879 resistant^*^	More resistant^*^	Not performed	n/a	Tested as treatment for advanced sarcoma trial ongoing (NCT00003718)
AGI-5198	Resistant^*^	Resistant^*^	Not performed	Poor PFS with IDH-1 inhibitor single-agent therapy (*n* = 3) (van Maldegem et al., 2019). Six month PFS rate of 53.8% and stable disease observed in 52% of patients (*n* = 13) (Tap et al., 2020)	n/a
ABT-737	Sensitive^*^ (van Oosterwijk et al., 2011)	SW1353 more resistant, CH2879, JJ012 more sensitive^*^	Not performed	n/a	n/a
Dox + ABT-737	Synergistic^*^ (van Oosterwijk et al., 2011)	Overall loss of synergy^*^	Not performed	n/a	n/a
YM-155	Sensitive^*^ (de Jong et al., 2016b)	More resistant^*^	Not performed	n/a	n/a
Sapanisertib	Sensitive^*^ (Addie et al., 2019)	CH2879 more sensitive, JJ012 and SW1353 more resistant^*^	Delayed tumor growth, development of resistance (Addie et al., 2019)	Unclear (Chawla et al., 2018; Trucco et al., 2018)	Combination of mTOR inhibition with cyclophosphamide/nivolumab recruiting (NCT02821507, NCT03190174)
Radiation	Resistant^*^ (de Jong et al., 2019; Venneker et al., 2019)	JJ012 more resistant, CH2879 and SW1353 comparable^*^	Not performed	In two retrospective studies: Advanced modality radiation treatment and higher dose treatment associated with improved survival in positive margin patient cohort (*n* = ?/680)^†^ (Catanzano et al., 2019). For patients who had locally advanced disease without metastases, radiotherapy was associated with a survival benefit (*n* = 45) (Van Maldegem et al., 2014). Proton therapy resulted in (*n* = ?/71)^†^ 94.1% 7-year OS in low-grade CS (Weber et al., 2016)	Advanced radiotherapy strategies for unresectable tumor trials recruiting using proton (NCT01449149) or stereotactic (NCT04098887) radiotherapy.

*Clinical trials, past results and ongoing studies, are also mentioned within the summary when applicable*.

* *Current paper*.

† *Unknown number of conventional CS subtype within cohort*.

We cannot exclude that the different growth rates of the various cell lines affect response in the spheroid model. For example, doxorubicin is known to preferentially affect proliferating cells (Yang et al., 2014), therefore this could be a contributing factor to the maintained sensitivity of CH2879 spheroids to this drug, since the amount of proliferation observed by immunohistochemistry was higher in this cell line. However, overall CH2879 growth, observed by presto blue assay, was comparable to JJ012 during the 72 h treatment period, yet the JJ012 spheroids were notably more resistant to doxorubicin than monolayer cultures (2D/3D 49.6). Therefore, growth rate may not be the only contributing factor.

Growth rate dependent variation between cell lines in 2D is commonly corrected for by using a metric such as the GR_50_. This metric relies on the assumption that the cells are in exponential growth before and during application of the drug, otherwise a reliable value cannot be achieved (Brooks et al., 2019). Applying these metrics to 3D cell culture models is more difficult, since these tend to have significantly slower growth rates. Therefore, in 3D, when cell growth rate is not high enough for GR_50_ calculation, differences between cell lines should still be considered separately, when observing the data by comparing the viability of control samples before and after the treatment period.

An advantage of the 3D alginate spheroid model is that in addition to monitoring cell viability, it enables morphological and immunohistochemical assessment after treatment. We previously used an siRNA screen and identified survivin as a potential target for treatment in chondrosarcoma (de Jong et al., 2016b). Here our previous results are confirmed as the survivin inhibitor YM-155 also reduced cell viability in the chondrosarcoma spheroids. However, while 2D results suggested a relation between p53 mutation status and response, this was not confirmed in the 3D spheroid model. The lack of a complete reduction in viability could also be due to reduced exposure of the cells to YM-155. Histological evaluation revealed that the spheroids which remained viable and proliferative were larger in size. Post-treatment spheroid colony number and size evaluation showed a reduced presence of smaller spheroids after treatment and no visible difference to larger spheroids. An increase in apoptosis was not observed after treatment with YM-155, which was consistent with previous data where we showed that YM-155 affected cell cycle progression. Previously published data on YM-155 sensitivity of a large panel of cancer cell lines indicated that resistance may also be associated with low SLC35F2 expression, therefore before moving onto *in vivo* testing expression of this efficacy biomarker could be studied within chondrosarcoma spheroids (Winter et al., 2014).

The anti-apoptotic Bcl-2 family members were earlier shown to be overexpressed and to play an important role in chemo resistance of chondrosarcoma (de Jong et al., 2018). Restoration of the apoptotic machinery, by inhibiting the Bcl-2 family members using ABT-737, induced apoptosis and reverted the resistance of chondrosarcoma cells to doxorubicin (van Oosterwijk et al., 2012b). In the present study, ABT-737 reduced cell viability and induced apoptosis in a majority of chondrosarcoma spheroids, although a limitation of this model is the presence of a moderate apoptotic signal in control samples. However, the synergy between doxorubicin and ABT-737 observed in monolayer cultures could not be confirmed in spheroids and IC_50_ values were increased in comparison to doxorubicin alone. By adjusting dosage amount and times, the synergistic effect could be improved. Therefore, in future experiments, synergistic effects should also be tested within spheroid models for a more representative result and refined dosing schedules.

A previously performed metabolic compound screen revealed an mTOR inhibitor, sapanisertib, as an effective anti-proliferative agent for chondrosarcoma. Reduction of oxidative and glycolytic metabolism and decreased proliferation was observed with treatment as well as a 5 day delay of tumor growth in a chondrosarcoma xenograft model (Addie et al., 2019). For comparison with xenograft results, a protocol for long-term drug treatment in spheroids was established. Reduced growth rate was observed between 1 and 7 days of treatment with 10 nM sapanisertib, in comparison to untreated controls, which then increased between 7 and 14 days in all cell lines, in line with *in vivo* data. A phosphokinase downstream of mTOR, pS6, was still expressed in spheroids after treatment, similarly to the xenograft model, suggesting the presence of a resistance mechanism. Previously published monolayer cultures, conversely, demonstrated inhibited pS6 expression after treatment with sapanisertib, which indicates that, in the context of the mTOR pathway, the spheroid model was more representative of *in vivo* results. Subsequently, mTOR inhibition would have a higher effectiveness as part of a combination treatment which would eliminate the remaining cell population (Boehme et al., 2018). In clinic, no significant single-agent mTOR treatment effect has been observed in chondrosarcoma patients thus far. A phase II study using ridaforolimus, another mTOR inhibitor, as a single agent did not have a noted effect on chondrosarcoma patients in the cohort (Chawla et al., 2018). However, prolonged stable disease was achieved for chondrosarcoma patients by combining mTOR inhibitors with liposomal doxorubicin or EGFR, IGF1R, VEGF inhibitors (Katz et al., 2016; Liu et al., 2016; Trucco et al., 2018; Vlahovic et al., 2018).

High hopes for effective therapy came with the identification of specific driver mutations in the isocitrate dehydrogenase genes IDH1 and IDH2, and the development of a specific inhibitor of mutant IDH1, such as AGI-5198. However, *in vitro* studies showed loss of clonogenic capacity only after long-term treatment with high concentrations (20 μM), while no effect was observed at lower concentrations or shorter treatment times (Li et al., 2015; Suijker et al., 2015). The spheroid model also did not show loss of viability due to IDH mutant inhibitor treatment. A more metabolically stable version of the inhibitor, ivosidenib (AGI-120), was approved for chondrosarcoma clinical trials (Popovici-Muller et al., 2018) and showed poor progression free survival with single-agent therapy in one retrospective study (van Maldegem et al., 2019), and more durable disease control in a phase I study, with 52% of patients experiencing stable disease. However, a placebo controlled randomized phase II study with saridegib (IPI-926) stopped for futility after an interim-analysis (Bussiness Wire, 2020). The company never released the data for a formal scientific publication.

The effect of radiotherapy on chondrosarcoma monolayer models varied between cell lines in several studies (de Jong et al., 2019; Venneker et al., 2019). A special protocol for a 3D version of the commonly used colony formation assay was developed using the alginate chondrosarcoma spheroid model to determine the effect of radiation on spheroid number and size. This method is not possible within all 3D models as some scaffolds, such as collagen-based ones, allow free migration of cells, preventing formation of individual colonies, and the ECM production capabilities of chondrosarcoma also make cell extraction from scaffolds difficult (Hamdi et al., 2015). JJ012 spheroids exhibited a higher resistance to radiotherapy than previously published 2D culture results (SF2 2D = 0.54/0.55, 3D = 0.83), whereas CH2879 showed similar survival (SF2 2D = 0.62, 3D = 0.58) and SW1353 maintained resistance to radiotherapy (SF2 2D = 1.00/0.88, 3D = 0.95) (de Jong et al., 2019; Venneker et al., 2019). Overall, all three spheroid models were substantially resistant to radiation, as is reported for chondrosarcoma in patients. Advanced radiotherapy modalities may be able to overcome the observed resistance, because radio resistance has been linked to the total radiation dose applied, and modern techniques enable the safe application of a higher total dose. Current clinical data indeed show that the use of advanced radiation modalities, such as intensity-modulated radiation therapy (IMRT), volumetric modulated arc therapy (VMAT), stereotactic radiosurgery (SRS) and especially particle therapy (radiotherapy with protons or carbon ions) result in a trend toward improved survival with higher radiation doses applied for patients with positive margins after resection (Weber et al., 2016; Catanzano et al., 2019). Therefore, the 3D chondrosarcoma spheroid model provides an excellent model to further investigate the use of these modalities for chondrosarcoma.

## Conclusion

Using a spheroid model of chondrosarcoma, representative of chondrosarcoma *in vivo* with regards to morphology and extracellular matrix production, we demonstrated short and long-term *in vitro* treatment strategies. This included assessment of synergistic effect and clonogenic capacity, in order to better predict *in vivo* response. Use of the alginate bead model for chemotherapeutic, radiotherapeutic and combination therapy testing would refine the selection of therapies for *in vivo* testing of chondrosarcoma. This approach is more efficient, cost effective, ethically beneficial, and may lead to discovery of novel treatment options for chondrosarcoma patients.

## Data Availability Statement

The raw data supporting the conclusions of this article will be made available by the authors, without undue reservation.

## Author Contributions

IP optimized 3D cell cultures and IP, SV, and BA set up 2D and 3D cultures for the study. IP, SV, and BA performed antineoplastic agent experiments. IP and AV performed data analysis of cell viability assays. HG and AK performed data analysis for clinical correlation. IB performed all IHC staining. IP analyzed 3D colony counts and IHC. JB conceived and supervised the study. IP and JB wrote the manuscript. All authors read and approved final manuscript.

## Conflict of Interest

The authors declare that the research was conducted in the absence of any commercial or financial relationships that could be construed as a potential conflict of interest.

## Abbreviations

2D: Two dimensional
3D: Three-dimensional
DMSO: Dimethyl sulfoxide
ECM: Extracellular matrix
FBS: Fetal bovine serum
GR50: Half maximal growth rate inhibitory concentration
H&E: Hematoxylin and Eosin
IC50: Half maximal inhibitory concentration
IDH: Isocitrate dehydrogenase
IMRT: Intensity-modulated radiation therapy
MCTS: Multicellular tumor spheroids
mut: mutant
PBS: Phosphate buffered saline
PBT: Proton-beam therapy
PFS: Progression-free survival
SF: Surviving fraction
SRS: Stereotactic radiosurgery
wt: wild type.
