# Supplementary material for: Selection of Effective Therapies Using Three-Dimensional in vitro Modeling of Chondrosarcoma
Source: Front Mol Biosci. 2020 Dec 21;7:566291. doi: 10.3389/fmolb.2020.566291 (PMC7793672; doi:10.3389/fmolb.2020.566291)
Supplement: Supplementary file 1 [file Data_Sheet_1.PDF]

Supplementary material

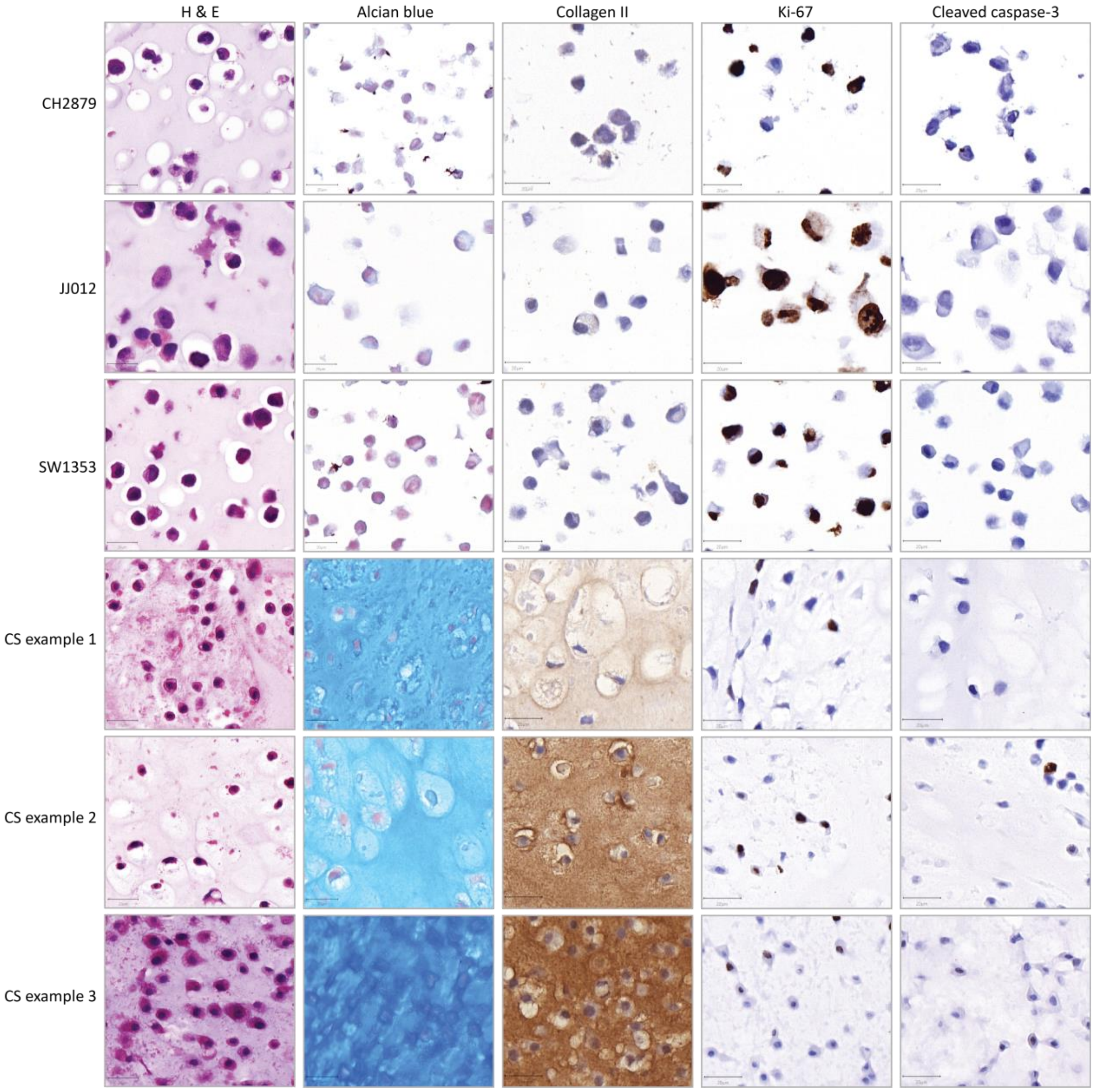

Figure S1. Histological and immunohistochemical stains of 2D cultured chondrosarcoma cell lines (CH2879, JJ012, SW1353) as well as three high grade human chondrosarcomas (CS example 1,2 and 3) demonstrate differences between 2D cultured cell lines and *in vivo.* The 2D cultures show lack of glycosaminoglycan production (alcian blue), lack of collagen II (IHC), overactive proliferation (Ki-67) and lack of apoptosis.

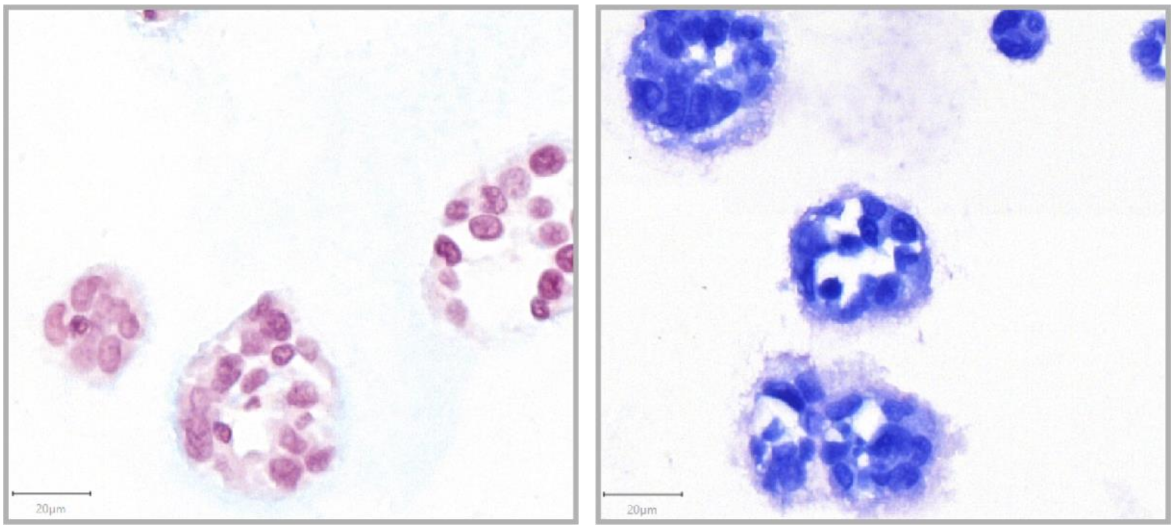

Figure S2. Alcian blue (left) and toluidine blue (right) stained SJSA-1 (osteosarcoma) spheroids, used as a negative control for the stains. Alcian blue positive stain= light blue and negative= only red nuclei. Toluidine blue positive stain= purple and negative= only blue nuclei. Scale bar= 20µm.

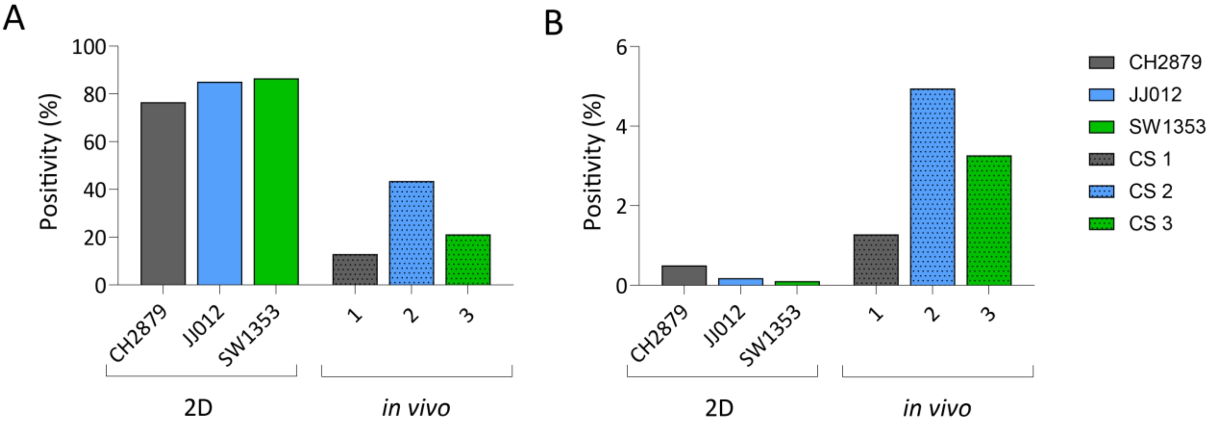

Figure S3. Quantification of proliferation (Ki-67) and apoptosis (cleaved caspase 3) shows differences between 2D cultured chondrosarcoma cell lines and human chondrosarcoma. A) Proliferation was observed in all cell lines at high levels (>77%) but was at much lower within tissues (<44%). B) Levels of apoptosis (cleaved caspase 3), were higher within tissue (>1.3%) than in 2D cultures (<0.5%) (n=1).

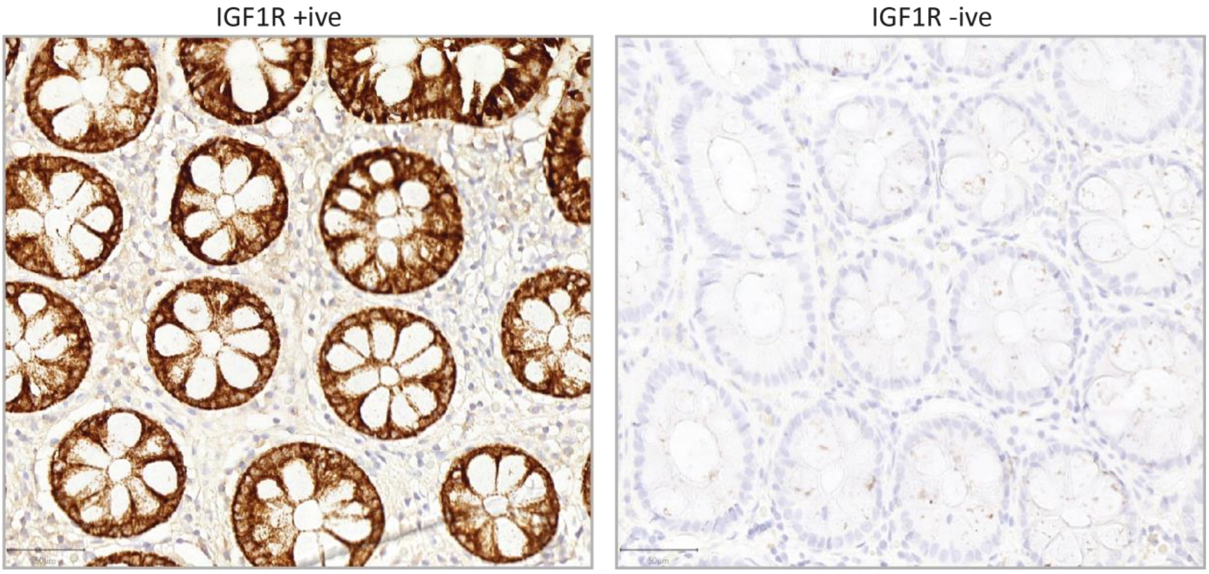

Figure S4. Colon tissue was used as a control for the IGF1R immunohistochemical stain. A clear difference between positive and negative controls can be observed.

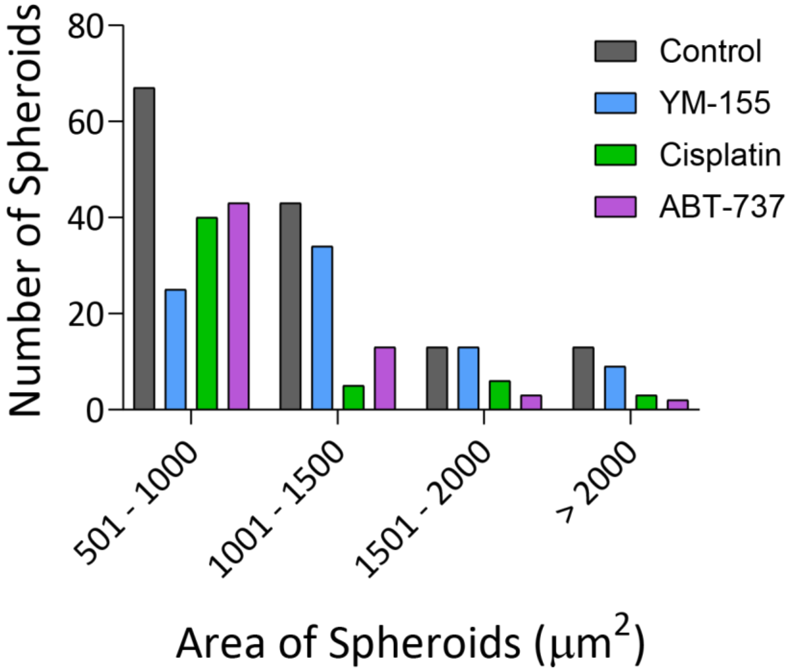

Figure S5. CH2879 spheroid colony number and size decrease with drug treatment. Overall size of spheroids decreases with treatment. Additionally, YM-155 treatment causes a decrease in spheroids of an area between 501-1000μm^2 (n=1).

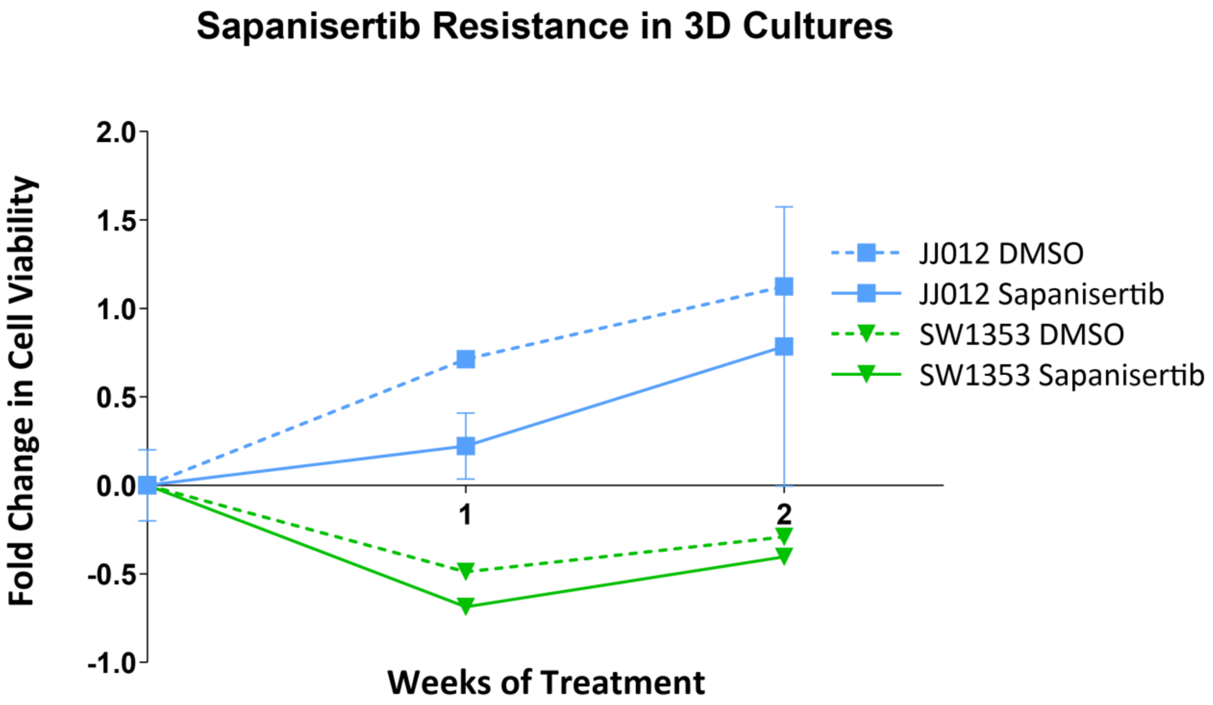

Figure S6. Long term treatment with sapanisertib (10nM) showed recovery of cell viability of JJ012 3D cultured cells treated with sapanisertib over 2 weeks. SW1353 cells experience an initial loss of viability regardless of treatment, however treated cells follow a similar trend to control cells (n=4).
